# A Criminologist’s View of the Divorce Question

**Published:** 1914-10

**Authors:** 


### A Criminologist's View of the Divorce Question.

Dr. Thomas Speed Mosby in "Causes and Cures of Crime," gives a most rational and conservative estimate of the divorce question as viewed by the modern eugenist.

"The question of divorce is a socio-religious question and is one that strikes directly at the heart of society. Sexual crimes are the specific crimes of advanced civilization, according to Lombroso, Penta, Viazzi and Krafft-Ebing; and Lombroso singles out the remedy of divorce as the most powerful preventive of such crimes. This conclusion appears to be based upon Ferri's statistics, which show that in Germany and France convictions for adultery and other sexual crimes, within a given period, were most numerous in districts where divorce did not exist. But the same statistics would as easily support an argument in favor of 'free love,' and the non-existence of marriage. The crime of adultery presupposes the existence of marriage. Abolish the marital relation and you preclude all possibility of violating it, of course; but such a course would suggest the experiment of the old Greek state in which there was no money coined save from iron which had first been rendered worthless—and this in order to prevent the people from loving money. Plato urging a community of wives and theoretical state paternity was not so inconsistent; but his premises were false. Divorce is not a panacea for sexual crimes, nor is it even a mild corrective. At least it has not been so with us. Assuredly, divorce is sufficiently prevalent in the United States. With us it amounts almost to a national scandal. But are sexual crimes less numerous here? The Chicago Vice Commission, in its report upon the social evil in Chicago (published in 1911), after an exhaustive consideration of the subject, records itself of the opinion that 'divorce, to a large extent, is a contributory factor to sexual vice.' Says August Drähms: 'Divorce and crime go hand in hand, and juvenile crime is sheltered beneath its wings.' Ireland, the least criminal nation of Europe, has the fewest divorces.

"After referring to the manner in which Tacitus contrasted the chastity of the German women with the conjugal infidelity of the Roman matrons, Gibbon, in the ninth chapter of Volume I in his history, observes: 'Although the progress of civilization has undoubtedly contributed to assuage the fierces passions of human nature, it seems to have been less favorable to the virtue of chastity, whose most dangerous enemy is the softness of the mind. The refinements of life corrupt while they polish the intercourse of the sexes. The gross appetite of love becomes most dangerous when it is elevated, or, rather, indeed, disguised, by sentimental passion. The elegance of dress, of motion and of manners, gives a lustre to beauty and inflames the senses through the imagination. Luxurious entertainments, midnight dances and licentious spectacles, present at once temptation and opportunity to female frailty.'

"Indeed, throughout all history, licentiousness has as certainly attended upon luxury as luxury has followed upon the heels of wealth. When the tide of materialistic ideas sets in upon a nation chastity is the first of the virtues to be set adrift. Will divorce stem the tide? There is reason to believe exactly the contrary. Fifty per cent of the children in our reform schools are the children of divorced parents. Fully 75 per cent of juvenile criminals have had bad home environment, and a very large proportion of adult criminals may trace their downfall to the ruined home.

"So long as we cannot guarantee infallibility in matrimonial selection the State must and will, under certain limitations and restrictions, recognize divorce. It may minimize, but not prohibit. However, the root of the evil lies not in separation, but in the causes which induce separation. Unhappy marriages are due to one or both of two general causes, viz: (1) hasty and inconsiderate marriage, and (2) improper conduct after marriage. Recognizing, no doubt, the dangers following from hasty marriages, Pope Innocent III, in the year 1215, established the publication of the marriage banns, and the marriage banns was afterwards recognized by the laws of England, in the statutes of George II and George III, as well as in the laws of some other nations. But the three weeks' notice thus provided is not sufficient in point of time, is not of universal application, and is not of sufficiently wide publicity. However, an extension of the idea involved in the banns would prevent many ill-judged marriages, and the subject is now receiving the consideration of many legislative bodies in the United States. * * * But after this has been said upon the subject of marriage and divorce, and sexual crime, we are brought to the conclusion that, after all, the home is the infallible standard and criterion of a nation's life. Influences that destroy the home, whatever they may be, will as certainly destroy alike the individual and the national character."

